# Loss of FoxA2 accelerates neoplastic changes in the intrahepatic bile duct partly via the MAPK signaling pathway

**DOI:** 10.18632/aging.102332

**Published:** 2019-11-05

**Authors:** Junyi Shen, Yongjie Zhou, Xiaoyun Zhang, Wei Peng, Chihan Peng, Qiang Zhou, Chuan Li, Tianfu Wen, Yujun Shi

**Affiliations:** 1 Department of Liver Surgery and Liver Transplantation Center, West China Hospital, Chengdu, China; 2 Laboratory of Pathology, Key Laboratory of Transplant Engineering and Immunology, MCH, West China Hospital, Sichuan University, Chengdu, China

**Keywords:** FOXA2, intrahepatic cholangiocarcinoma, prognosis, TAA, MAPK signaling pathway

## Abstract

Background: Intrahepatic cholangiocarcinoma (ICC) is characterized by a highly aggressive nature and a dismal outcome. FOXA2 is an archetypal transcription factor involved in cholangiocyte proliferation.

Results: FOXA2 expression was negatively correlated with tumor stage (p = 0.024). Univariate and multivariate analyses showed that low FoxA2 expression was associated with tumor relapse and survival. At 20 weeks after TAA administration, FoxA2^-/-^ mice displayed significant manifestations of neoplasia, while WT mice did not.

RNA sequencing analysis showed that the expression of genes in the MAPK signaling pathway was significantly higher in FoxA2^-/-^ mice. IHC and Western blot results showed that p-ERK1/2, CREB1 and RAS were highly expressed in FoxA2^-/-^ mice. Furthermore, using in vitro experiments with siRNA, we found that low expression of FoxA2 could exacerbate the metastatic potential of ICC. The expression of p-ERK1/2 and RAS, which are key mediators of the MAPK signaling pathway, was significantly increased.

Conclusion: Low FOXA2 expression negatively affected the prognosis of patients with ICC. Loss of FoxA2 expression could promote intrahepatic bile duct neoplasia partly via activation of the MAPK signaling pathway.

Materials and methods: In all, the data of 85 patients with ICC were retrospectively collected and analyzed. TAA was used to induce ICC in FoxA2^-/-^ mice and WT mice. RNA-sequencing analysis was used to identify the expression of different genes.

## INTRODUCTION

Intrahepatic cholangiocarcinoma (ICC) is the second most common primary hepatic malignancy, as it accounts for 5%-10% of all primary liver cancers [1]. Surgical resection is the most widely used and potential radical treatment for ICC. The 5-year recurrence rate is as high as 60%, and the survival rate is only 21%-35% [2, 3]. Given its highly aggressive nature and dismal outcome, it is of great value to investigate the mechanisms of the occurrence and development of ICC and to identify potential molecular targets for treatment. The occurrence of ICC is mainly related to inflammatory reactions and the transcription of a large number of reprogramming genes, which are associated with the gradual loss of substantial functional gene expression and the progressive activation of oncogenes [4]. The administration of thioacetamide (TAA), as a potent hepatotoxin and carcinogen, can contribute to bile duct cell proliferation and can eventually lead to the establishment of a cholangiocarcinoma model [5].

During the transformation process, key transcription factors play a critical role [4]. Forkhead box transcription factor A2 (FOXA2) is a liver-enriched transcription factor that specifically binds to the promoter positions of three liver-specific genes, namely, transthyretin, α1-antitrypsin and albumin [6, 7]. Recently, FOX family members have been determined to be involved in the regulation of more than 50% of functional genes in the liver, and these proteins were also found to be related to liver development, glycolipid metabolism and cholangiocyte proliferation [8, 9]. Liver-specific knockdown of FOX family members can affect the transcription of the bile acid transporter gene, which leads to intrahepatic cholestasis [10]. FoxA2 can regulate differentiation and proliferation of biliary cells during fetal liver development [11, 12]. The stable expression of FoxA2 can regulate biliary-committed progenitor cells, which exert therapeutic effect in bile ducts in individuals with cholestatic liver injury [12]. A high level of FoxA2 expression might exert protective effects in the context of liver injury [12, 13].

Notably, previous studies reported that the dysfunction of FOXA2 is related to the prognosis of several cancers [14–17]. FOXA2 can inhibit the transcription of metalloproteinase-9 (MMP-9) and can attenuate the invasion and metastasis of tumor cells [16]. Moreover, this protein can inhibit epithelial to mesenchymal transition, thus abolishing cancer metastasis [15, 18, 19]. The decrease in FOXA2 expression could widely influence tumor-related signaling pathways, and can thus influence tumor prognosis [20, 21].

ICC is essentially the result of uncontrolled proliferation and malignant transformation of bile duct cells caused by an injury stimulus. As a key transcription factor that regulates the proliferation and function of bile duct cells, the role of FoxA2 expression in the development and prognosis of ICC remains unclear.

## RESULTS

### Low expression of FoxA2 expression is a risk factor in ICC patients

**Figure 1:**
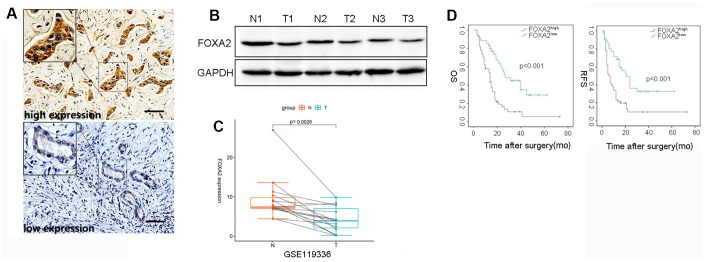
**The correlation between intratumor FOXA2 expression and outcomes.** (**A**) Representative immunostaining images of FOXA2 in ICC; (**B**) Protein level of FOXA2 in adjacent noncancerous tissue and tumor tissue. GAPDH was used as the loading control; (**C**) Paired analysis showed that FOXA2 expression was decreased in tumor samples. T: tumor tissue; N: adjacent normal tissue; (**D**) The overall survival (OS) and recurrence-free survival (RFS) rates of 85 ICC patients were compared between the low- and high-FOXA2 expression groups (P < 0.001, log-rank test).

To assess the clinical significance of FoxA2 expression in ICC, immunohistochemistry for FoxA2 was performed in tumor tissue (n=91). The IHC result revealed that 35 ICC patients had low FOXA2 expression and that 50 ICC patients had high FOXA2 expression. Representative immunostaining images (high and low) of FOXA2 are presented in Figure 1A. Compared with adjacent tumor tissue, the expression of FoxA2 was decreased in ICC tissues according to the Western blot analysis (Figure 1B). Interestingly, according to the RNA-seq results of 15 pairs of ICC tumors and matched nontumor liver tissues, FOXA2 was significantly downregulated in the paired tumor samples (Figure 1C, p=0.0028).

As shown Table 1, the majority of clinicopathological parameters were similar in both groups. Notably, the expression levels of FoxA2 were inversely correlated with tumor grade. A univariate analysis showed that tumor size (p = 0.05), vascular invasion (p = 0.032), FoxA2 expression (p <0.001), positive margins (p = 0.004), lymph node metastasis (p <0.001) (P = 0.005), AJCC stage (p < 0.001), PLT (p = 0.003), and ALB (p = 0.007) were associated with relapse. The results of the multivariate analysis showed that FoxA2 expression (low vs high, HR2.772, 95% CI 1.573-4.886, p <0.001), positive surgical margins (HR = 6.1, 95% CI 2.145-17.347, p = 0.001), lymph node metastasis (HR = 2.207, 95% CI 1.550-4.454, p = 0.001), satellite lesions (HR = 2.368, 95% CI 1.296-4.328, p = 0.005), and ALB (HR = 0.933, 95% CI 0.892-0.933, P = 0.002) were associated with tumor relapse (Table 2). The survival analysis (Figure 1D) showed that the tumor-free survival rates at 1 year and 3 years in the high FOXA2 expression group were 68.6% and 32.9%, respectively, and were 34.0% and 9.9%, respectively in the low FOXA2 expression group (p < 0.001). The overall survival rate at 1 year and 3 years in the high FOXA2 expression group was 58.7% and 44.8%, respectively, and was 58.0% and 10.9%, respectively, in the low FOXA2 expression group (p <0.001). Therefore, low FOXA2 expression in ICC may indicate a higher tumor stage and dismal clinical outcome.

**Table 1:** Clinical characteristics of patients grouped by FoxA2 expression.

	**FoxA2^high^(n=50)**	**FoxA2^low^(n=35)**	**p value**
Age	55±13.1	57±10	0.59
Gender(M/F)	25/25	17/18	0.897
Positive HBsAg	19(38.0%)	7(20.0%)	0.096
Liver cirrhosis	9(18.0%)	5(14.3%)	0.771
Tumor size(cm)	6.7±3.2	6.0±1.9	0.277
Tumor number(single/multiple)	1/49	5/30	0.077
Differentiation(poor/moderate-well)	38/12	23/12	0.335
Vascular invasion	10(20%)	4(11.4%)	0.38
Bile duct thrombus	2(4.0%)	1(2.9%)	1
Nerve invasion	3(6.0%)	3(8.6%)	0.687
Positive surgical margin	4(8.0%)	2(5.7%)	1
MVI	7(14.0%)	4(11.4%)	1
Lymphonode involvement	13(26.0%)	4(11.4%)	0.098
Local invasion	8(16.0%)	3(8.6%)	0.514
Satellite lesion	12(24.0%)	6(17.1%)	0.591
Elevated serum CA19-9	36(72.0%)	19(54.3%)	0.093
The AJCC staging system(early/late)	20/30	6/29	0.024
Platelet count(*10^9)	172±70.4	161±70.9	0.481
TIBL(umol/l)	13.7±5.4	17.2±29.2	0.409
ALT(U/L)	36.5±41.1	33.1±29.2	0.68
ALB(g/l)	41.7±5.0	42.2±5.7	0.661
Lymphocyte count	1.4±0.5	1.5±0.6	0.527

HBsAg: hepatitis B virus surface antigen; MVI: microvascular invasion; CA19-9: Carbohydrate antigen 19-9; TIBL: total bilirubin; ALT: alanine aminotransferase; ALB: albumin

**Table 2:** Univariate and Multivariate Analysis for prognosis of ICC patients after surgery.

	**Univariate**			**Multivariate**		
	**p value**	**HR**	**95%CI**	**HR**	**95%CI**	**p value**
Age	0.218					
Gender(M/F)	0.518					
Positive HBsAg	0.267					
Liver cirrhosis	0.357					
Tumor size(cm)	0.05					
Tumor number(single/multiple)	0.061					
Differentiation(poor/moderate-well)	0.198					
Vascular invasion	0.032	1.964	1.056-3.643			
FoxA2 expression(Low vs. High)	<0.001	2.865	1.666-4.929	2.772	1.573-4.886	<0.001
Positive surgical margin	0.004	4.102	1.583-10.628	6.1	2.145-17.347	0.001
MVI	0.099					
Lymphonode involvement	<0.001	3.22	1.765-5.876	2.907	1.550-4.454	0.001
Local invasion	0.17					
Satellite lesion	0.003	2.738	1.336-4.230	2.368	1.296-4.328	0.005
Elevated serum CA19-9	0.005	2.27	1.281-4.021			
The AJCC staging system(early/late)	<0.001	2.607	1.523-4.463			
Platelet count(*10^9)	0.003	1.006	1.002-1.010			
TIBL (umol/l)	0.836					
ALT(U/L)	0.287					
ALB(g/l)	0.007	0.948	0.912-0.985	0.933	0.892-0.933	0.002
Lymphocyte count	0.911					

### Loss of FOXA2 promotes intrahepatic bile duct proliferation and neoplasia

**Figure 2:**
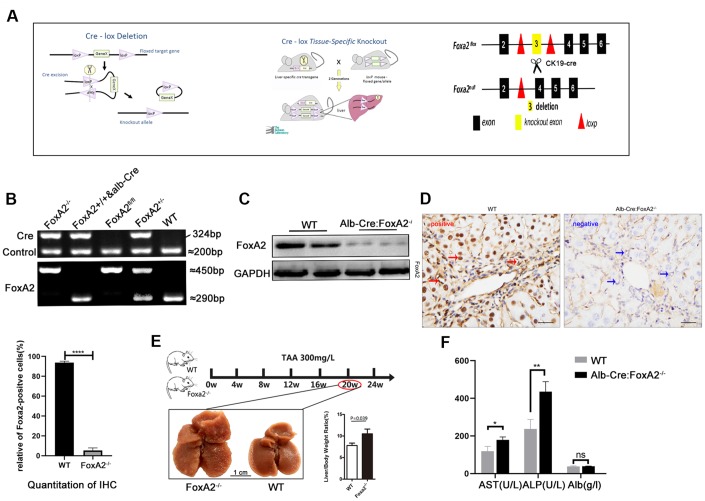
**Establishment of the TAA-induced cholangiocarcinoma model.** (**A**) Diagram of the DNA insertion site in the loxP alleles of FoxA2. The activation of albumin expression led to the abolishment of FoxA2 expression; (**B**) Electropherogram of tail DNA genotyping; (**C**, **D**) Western blotting and immunohistochemistry were performed to confirm the deletion of FoxA2 expression; (**E**) Gross observation of liver tissues at the indicated time points (Scale bar: 1 cm). Multiple nodes were detected in FoxA2^-/-^ mice, and the liver / body weight ratio was significantly higher in FoxA2^-/-^ mice; (**F**) The ALP and ALT levels were higher in FoxA2^-/-^ mice based on the serum biochemistry test. ALP: alkaline phosphatase; ALT: Alanine amino-transferase; ALB: albumin.

To determine its clinical significance and the role of FoxA2 in cholangiocyte proliferation [11], Alb-Cre mice were mated with FoxA2^loxp/loxp^ mice to generate liver-specific FoxA2 knockout mice (Alb-Cre;FoxA2^-/-^) (Figure 2A). The genotypes of the wild-type mice and knockout mice were confirmed by PCR of tail DNA (Figure 2B). Furthermore, IHC showed a significant reduction in FoxA2 expression levels. Western blot showed that FoxA2 expression in FoxA2 knockout mice was significantly decreased (Figure 2C and 2D). Compared with WT mice, no obvious abnormalities were observed in body weight, behavior or liver tissues.

Previous studies have shown that drinking water containing TAA at 300 mg/L could induce cholangiocarcinoma in the liver. After 20 weeks, liver cirrhosis had begun to develop [5]. To investigate the role of FoxA2 in the development of ICC, we harvested the liver and blood from both groups after 20 weeks. We found that liver/body weight was higher in the Alb-Cre: FoxA2^-/-^ mice compared with wild type mice (Figure 2E). The serum albumin level was observed to be similar in both groups, and ALP and AST levels were elevated in FoxA2^-/-^ mice (Figure 2F).

**Figure 3:**
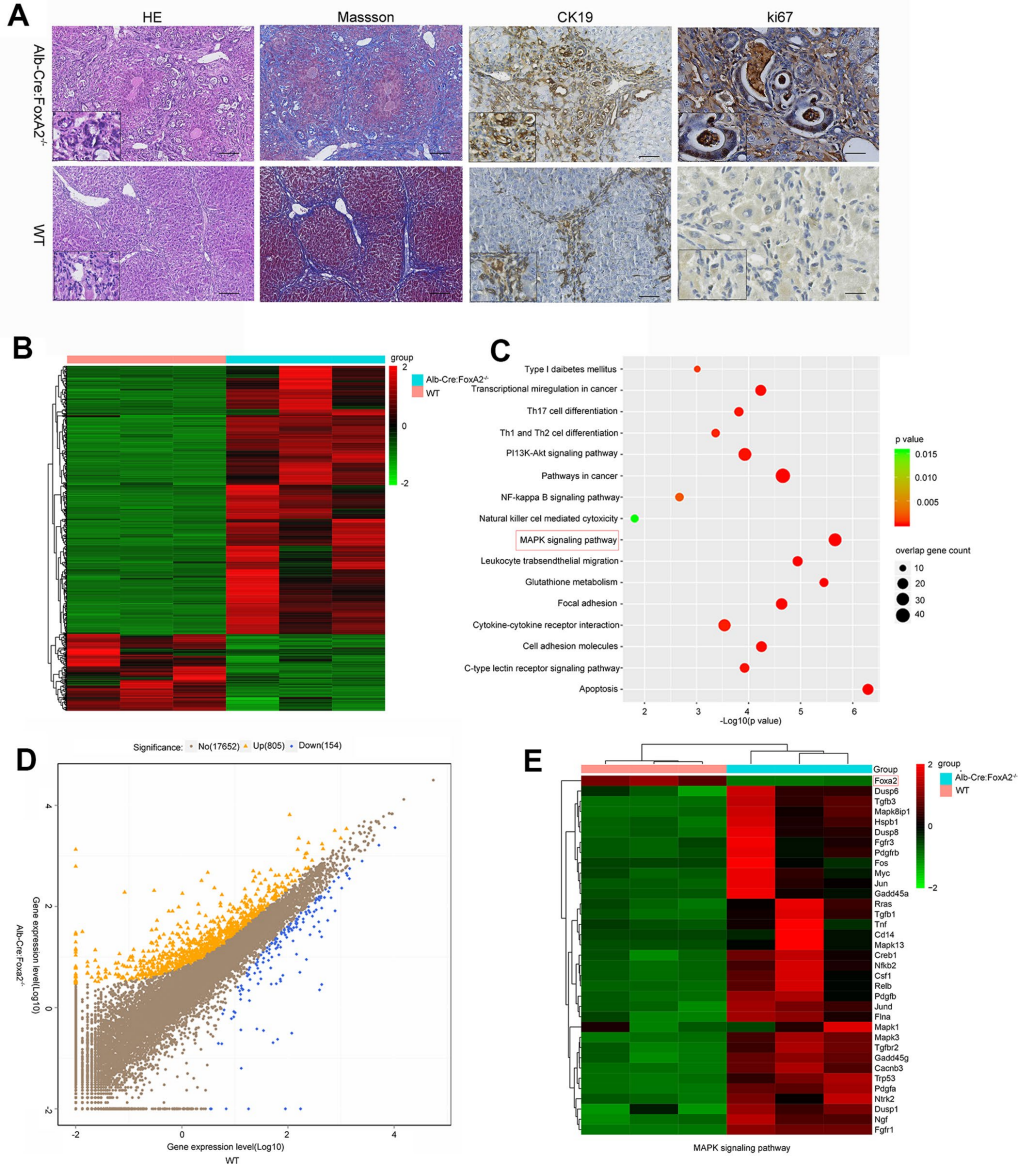
**Loss of FoxA2 promotes the development of intrahepatic bile duct neoplasms and enhances MAPK-related gene expression.** (**A**) Hematoxylin and eosin staining showed remarkable bile duct neoplasm formation in FoxA2^-/-^ mice. Significant liver cirrhosis and proliferation (Ki67 immunohistochemistry) were also observed in the bile duct in FoxA2^-/-^ mice (n=3 each group); (**B**) Heatmap showing the most differentially expressed genes (DEGs) between FoxA2^-/-^ mice and WT mice. (n = 3 samples per group); (**C**) KEGG analysis of biological processes. Most of these DEGs were clustered in the “MAPK signaling pathway” category, followed by the “Pathways in cancer”, and “PI13K-AKT signaling pathway”, and so on. The bar indicates the P value; the threshold of P = 0.015 is shown; (**D**) Volcano plot of P values as a function of the weighted fold change for mRNAs; 805 genes were upregulated genes and 154 were down regulated in FoxA2^-/-^ mice compared with WT mice.; (**E**) the activation of MAPK-signaling-related genes expression in FoxA2^-/-^ mice.

Masson staining suggested that both groups developed liver cirrhosis. Interestingly, at 20 weeks after TAA administration, H&E (hematoxylin and eosin) staining revealed that the loss of FoxA2 resulted in early and profound dysplastic changes in the biliary epithelium, which subsequently progressed to biliary cytokeratin (CK19)-expressing invasive ICC. However, in WT mice, the dysplastic changes in the biliary epithelium were mild. The TAA-induced bile duct neoplasia in FoxA2^-/-^ mice displayed typical ICC features, including abnormal proliferating bile duct cells, enlarged nuclei, loss of nuclear polarity and focal expression of CK19 [5]. IHC analysis was used to validate the depletion of FoxA2 from the liver of FoxA2^-/-^ mice, while Ki67 staining indicated the active proliferation of bile duct cells in FoxA2^-/-^ mice (Figure 3A).

### Loss of FoxA2 is related to activation of the MAPK signaling pathway in ICC development

To identify the molecular mechanisms underlying the growth effects caused by FoxA2 knock out, transcriptomic analysis using gene expression microarrays was performed in liver tissues from TAA-induced FoxA2^-/-^ mice and WT mice after 20 weeks. In terms of differentially expressed genes, 805 were upregulated and 154 were downregulated (Figure 3B and 3D). KEGG pathways with P-values < 0.05 were regarded as significant. As shown in Figure 3C, tumor-related signaling pathways, such as the MAPK signaling pathway, PI3K-AKT signaling pathway and NF-кB signaling pathway, were significantly enriched by these genes.

**Figure 4:**
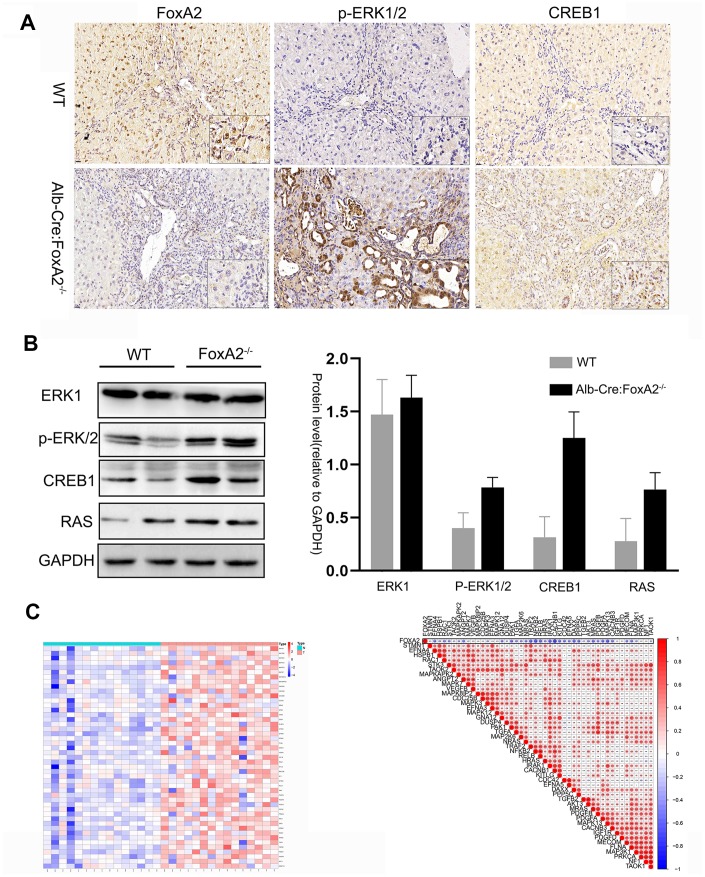
**The key components of the MAPK signaling pathway were activated in FoxA2^-/-^ mice.** (**A**, **B**) IHC and Western blotting analysis of low FoxA2 and high p-ERK1/2, CREB1 and RAS expression in FoxA2^-/-^ mice; © The heatmap shows the activation of key genes in the MAPK signaling pathway in ICC patients. These genes were selected from the KEGG database for MAPK signaling pathways (Supplementary Table 1); (**D**) The correlation plot shows that low FOXA2 expression was correlated with high expression of genes in the MAPK signaling pathway in ICC patients.

A previous study revealed the role of the MAPK signaling pathway in increasing the efficiency of hepatocyte differentiation [22]. Some studies suggested a relationship between the MAPK signaling pathway and FoxA2 [23]. In the current study, we selected the genes in the MAPK signaling pathway to perform a differential analysis. Interestingly, we found that the MAPK signaling pathway was highly activated in the FoxA2^-/-^ mice. As shown in Figure 3E, depletion of FoxA2 led to the activation of the MAPK signaling pathway in the bile duct in FoxA2^-/-^ mice treated with TAA for 20 weeks. Consistently, IHC and Western Blot results showed that FoxA2 expression was significantly decreased in the livers of FoxA2^-/-^ mice compared with WT mice. Notably, the phosphorylation of ERK1/2 and CREB1 was also markedly increased in the dysplastic bile ducts of FoxA2^-/-^ mice (Figure 4A and 4B).

Zhou G et al. performed RNA-seq in 15 pairs of ICC tumors and matched nontumor liver tissues using an Illumina HiSeq2000. We reanalyzed the expression of the FOXA2 gene and found its expression was downregulated in tumors. We then selected the key components in the MAPK signaling pathway. Interestingly, as the result of the heatmap analysis, shows the MAPK signaling pathway was activated in ICC tumor samples compared with paired normal samples (Figure 4C). Moreover, the expression of these key mediators was negatively correlated with FOXA2 expression (Figure 4D).

### Suppression of FOXA2 inhibits the malignant phenotype of tumor cells in vitro

**Figure 5:**
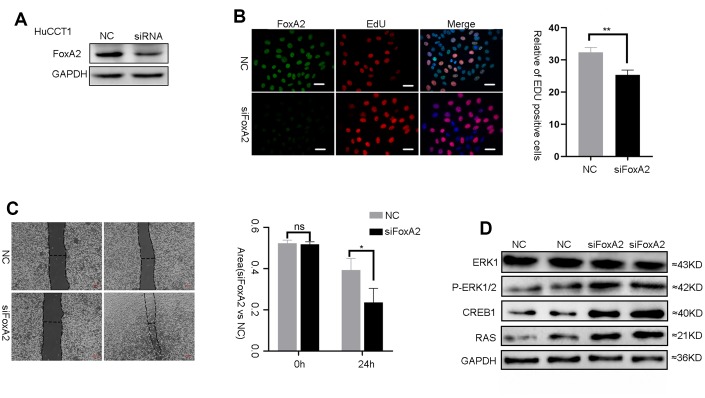
**The suppression of FoxA2 promotes activation of the MAPK signaling pathway.** (**A**) The effect of FOXA2 suppression in HuCCT1 cells by siFOXA2; (**B**) Cell proliferation was detected with EdU in ICC cells after suppression of FoxA2; (**C**) The invasion assay was used to assess the effects of FoxA2 down regulation on ICC cell invasion; (**D**) The expression of ERK1, P-ERK1/2, CREB1 and RAS in ICC cell lines was detected by Western blot after the suppression of FoxA2.

FoxA2 is a tumor suppressor in HCC and breast cancer [16, 18]. However, the role of FoxA2 in ICC requires further investigation. To evaluate the effect of FoxA2 on the malignant phenotype of ICC cells, FoxA2 expression was downregulated in HuCCT1 cells using siFoxA2 (Figure 5A). Downregulation of FoxA2 using siFoxA2 promoted the growth of HCC cells. As shown in Figure 5B, the number of ICC cells that incorporated EdU in the siFoxA2 group was higher than that in the control group. We next performed a wound-healing assay to evaluate the metastatic potential of HuCCT1 cells (Figure 5C). The results showed that siFoxA2 exacerbated the metastatic potential of these cells. Consistently, the expression of p-ERK1/2, CREB1 and RAS was significantly activated by the decrease in FoxA2 expression in HuCCT1 cells. (Figure 5D). Taken together, these results implied that the loss of FoxA2 might promote the malignant phenotype of ICC via activation of the MAPK signaling pathway.

## DISCUSSION

In this study, we found that the expression of FOXA2 was significantly decreased in intrahepatic cholangiocarcinoma (ICC) in 41.2% of all patients. Consistently, data from expression profiling by high-throughput sequencing suggested that FOXA2 mRNA was significantly lower in tumor samples compared with paired noncancerous tissue (GSE119336, Provided by Zhou G, Cao P and Li Y). These results suggested that FOXA2 might play role in ICC development. Further, patients with ICC were divided into subgroups according to FOXA2 expression. Interestingly, the 3-year recurrence-free survival and overall survival rates in patients with high FOXA2 expression were significantly better than rates in patients with low FOXA2 expression (RFS: 32.9% vs. 9.9%; OS: 44.8% vs. 13.6%). FOXA2 expression was identified to be an independent risk factor for prognosis according to the multivariate analysis (HR: 2.72, p<0.001). Consistently, previous studies reported that in breast carcinoma and gastric cancer, patients with higher FOXA2 expression had a better prognosis [14, 17]. FOXA2 might act as a suppressor of tumor metastasis via regulation of epithelial to mesenchymal transition or tumor-related signaling pathways [15, 16] Low FoxA2 expression was found to be positively correlated with higher tumor stage (p=0.032), which indicates the negative relationship between FOXA2 expression and advanced tumor stage. Additionally, lymph node status, surgical margins, and satellite lesions have demonstrated to be independent risk factors among patients with ICC and were also identified by other investigators [24, 25]. Notably, the presence of lymph node metastasis was not uncommon in ICC patients (up to 26.0%) and could determine the prognosis of ICC patients. Despite the lack of statistical significance between both groups, the low FOXA2 expression group seemed to have a higher rate of lymph node metastasis (26.0 vs. 11.4%).

FOXA2 is an archetypal transcription factor in liver specification as it plays an indispensable role in bile duct cell differentiation, proliferation and tissue regeneration [12]. Currently, very few studies have investigated the role of FOXA2 expression in intrahepatic cholangiocarcinoma. The absence of FoxA2 does not lead to the spontaneous development of ICC despite that the depletion of FoxA2 in the endoderm stage can lead to death in mice [26]. Previous studies have shown that the oral administration of 300 mg/l TAA could induce cholangiocarcinoma over a prolonged time and that the earliest incidence of liver fibrosis begins at 20 weeks [5]. We constructed FoxA2 conditional-knockout mice using the same strategy [27] and generated a TAA-induced intrahepatic cholangiocarcinoma model. In the current study, drinking water containing 300 mg/l TAA was administered orally to both the FoxA2^-/-^ and wild type mice. To study whether the loss of FoxA2 promotes the early-stage development of ICC, we primarily focused on the difference between both groups at 20 weeks.

Signs of liver cirrhosis were observed in both groups. Biliary cytokeratin (CK19) expression within the intrahepatic portal area was also evident but was much higher in the FoxA2^-/-^ mice. Similarly, Ki67 expression was conspicuous in the proliferating bile duct cells of FoxA2^-/-^ mice. These findings indicated that the loss of FoxA2 expression might promote cholangiocyte proliferation under hepatotoxicant injury. Remarkably, we found that the changes in histologic features were more obvious in FoxA2^-/-^ mice and included demonstrable intrahepatic bile duct atypia, enlarged nuclei and loss of nuclear polarity. We also found that the liver/body weight ratio was higher in FoxA2^-/-^ mice, which suggested severe liver injury and regeneration.

The level of ALP, which is an indicator of inflamed bile ducts, was much higher in FoxA2^-/-^ mice. This indicated that loss of FoxA2 in bile duct cells might contribute to intrahepatic bile duct inflammation in TAA-induced chronic hepatocellular injury. Kelly McDaniel et al study suggested that biliary-committed progenitor cells that are regulated by FoxA2 are more resistant to hepatobiliary injury [12]. Stable FoxA2 expression might protect cells from hepatotoxins and carcinogens and thus may prevent carcinogenesis.

To investigate the underlying mechanism of FoxA2, we performed an RNA sequencing analysis. The results showed that 805 genes were upregulated and that 154 genes were downregulated. KEGG pathway analysis suggested that the MAPK signaling pathway was the pathway that was most correlated with ICC development in our model. As the heatmap shows, the key genes related to the MAPK signaling pathway were significantly upregulated, while FoxA2 expression was significantly decreased in FoxA2^-/-^ mice. As previous reports showed, the MAPK signaling pathway extensively participates in cell proliferation and differentiation and is related to tumor prognosis including in intrahepatic cholangiocarcinoma [28–30]. Consistently, the results of the IHC analysis suggested that the expression of key mediators in the MAPK signaling pathway, such as p-ERK1/2 and RAS, was significantly increased. We also knocked down FOXA2 mRNA expression by siRNA and again confirmed that suppression of FOXA2 in intrahepatic cells could promote cell proliferation and invasion. Western Blot analysis showed that p-ERK1/2 and RAS were highly expressed in cancer cells in which FOXA2 was inhibited by siRNA. These proteins have been shown to be closely related to tumor prognosis and to play a critical role in MAPK signaling [31–33].

We also observed activation of the MAPK signaling pathway in ICC in a cohort from the GEO database. Consistently, activation of the MAPK signaling pathway in cholangiocarcinoma was also demonstrated in previous studies [34, 35]. Furthermore, a negative correlation between FoxA2 expression and activated genes in the MAPK signaling pathway was observed. Low FoxA2 expression was negatively correlated with high expression of genes in the MAPK signaling pathway. On the contrary, tumor-related signaling pathways such as the PI3K/AKT and NF-kB signaling pathways as well as TH17 cell activation, have been investigated and shown to be closely related to biliary cancers [36–38]. The KEGG dataset showed that CREB was regulated by the AKT signaling pathway (Supplementary Figure 1). In the current study, the result of KEGG pathway analysis also revealed activation of the ATK signaling pathway. CREB1 was also increased in FoxA2^-/-^mice. The results from the RNA sequencing analysis showed that these signaling pathways might interact with FOXA2 expression and promote ICC development.

The current study has some limitations. The Alb-Cre: FoxA2^-/-^ mice presented with the loss of FoxA2 expression in biliary epithelia and hepatocytes. Given that in normal conditions, the biliary epithelia comprise only 3% of the total cellular population, the role of FoxA2 in TAA-induced ICC might be diluted. However, we performed in vitro experiments on ICC cells. We knocked down FoxA2 expression by siRNA and demonstrated that the decrease in FoxA2 expression in ICC cells could similarly promote tumor cell proliferation and invasion.

## CONCLUSIONS

For the first time, our study demonstrates that FOXA2 is independently associated with the prognosis of ICC patients after surgery. The loss of FoxA2 accelerated ICC development partly via the MAPK signaling pathway. FoxA2 may therefore be a potential target for ICC therapy.

## MATERIALS AND METHODS

### Human tumor tissues and follow up

Tumor samples were obtained from West China Biobanks, Department of Clinical Research Management, West China Hospital, Sichuan University (Chengdu, China). Written informed consent was obtained from all patients. All patients were pathologically diagnosed ICC by pathologists in west china hospital. Immunohistochemistry (IHC) of tissue slides was performed using an anti-FOXA2 antibody. FOXA2 expression was assessed using a four-point scale (negative, 1; weak positive, 2; positive, 3; strong positive, 4), according to the percentage of stained cells using Image-scope software (Aperio Technologies). Point 1 or 2 was classed into FOXA2^low^ group, and point 3 or 4 was classed into FOXA2^high^ group, respectively. Gene expression data of paired ICC patients were downloaded from NCBI Gene Expression Omnibus (http://www.ncbi.nlm.nih.gov/geo/) by GSE119336.

All patients were followed up at the first, third and sixth months in the first half year after the operation, every 3 months throughout the following 3 years and every 6 months thereafter after surgery. Recurrence free survival (RFS) was defined as the interval between the date of surgery and the detection of tumor recurrence (intrahepatic recurrence or/and extrahepatic metastasis), and overall survival (OS) was defined as the interval between the date of surgery and death.

### Animal experiments

The experiments on animal were conducted in accordance with national and international laws and policies and approved by the Animal Care and Use Committee of Sichuan University. FoxA2^loxP/loxP^ mice were intercrossed with Albumin-Cre transgenic mice to obtain constitutive (Alb-Cre:FoxA2^−/−^) liver-specific FoxA2- deficient mice. Littermates without Cre were used as wild type controls (WT). All the mice used were fed a normal chow diet under SPF conditions. The animals were divided into two groups, including WT group and an experiment group. The experiment group (FoxA2^-/-^ mice) and control group (WT mice) were administered Thioacetamide (TAA) 300 mg/l in their drinking water every day up to the time they were killed. the animals were weighed weekly to calculate their body weight gain. Furthermore, blood samples were drawn from biochemistry tests: albumin, alanine aminotransferase (ALT), alkaline phosphatase (ALP) were determined using the standard techniques.

### Western blotting analysis and immunohistochemistry

Proteins were extracted using RIPA buffer with protease inhibitor cocktail. Western blot was performed using standard protocols. The antibodies used in this study were as follows: FOXA2 (1:1000, ab5074, Abcam, UK). ERK1(1:1500, Cat#: ET1604-32, Hangzhou HuaAn Biotecchnology, China), ERK1(pT202/pY204)+ERK2 (pT185/pY187)(1:1500, Cat#: ET1610-13, Hangzhou HuaAn Biotecchnology, China), CK19(1:1500, Cat#: ET1610-13, Hangzhou HuaAn Biotecchnology, China), RAS(1:1500, Cat#: ER40115, Hangzhou HuaAn Biotecchnology, China), CREB1(1:1500, Cat#: ET1601-15, Hangzhou HuaAn Biotecchnology, China)

Liver specimens were harvested and fixed with 10% buffered formalin for 48 h and then processed for sectioning by embedding in paraffin. Staining with hematoxylin and eosin and immunohistochemical analyses was conducted using standard protocols. The antibodies used in this study are as follows: FOXA2(1:100, ab5074, Abcam, UK), CK19 (1:1 00, ab133496, Abcam, UK), ERK1(pT202/pY204)+ERK2 (pT185/pY187)(1:100, Cat#: ET1610-13, Hangzhou HuaAn Biotecchnology, China), CREB1(1:100, Cat#: ET1601-15, Hangzhou HuaAn Biotecchnology, China), KI67(1:200, ab16667, Abcam, UK). 3 paired FoxA2^-/-^ mice and WT mice were used for the analysis of gene expression.

### Cell culture, transfection, cell proliferation and invasion

The ICC cell line HuCCT1 was obtained from the Institute of Biochemistry and Cell Biology (Chinese Academy of Sciences, Shanghai, China). The cells were cultured in Dulbecco’s Modified Eagle’s Medium containing 10% heat-inactivated fetal calf serum. The small interfering RNA (siRNA) against FoxA2 (the target sequence: CCATGAACATGTCGTCGTA) was synthesized by RiboBio (Shanghai, China). Tumor cells (3 *10^3^ cells/well) were seeded in 96-well plates and were allowed to grow for 24 h. Primary human intrahepatic cholangiocarcinoma cells were transfected with siRNA or NC for 72 h. A 5-Ethynyl-2′-deoxyuridine (EdU) assay to assess cell proliferation was performed according to the manufacturer’s instructions. The result was analyzed using the mean number of cells in three fields for each sample. To assess the invasion ability of tumor cells, a wound healing assay was performed. Cells were grown in a 6-well plate, and after they reached confluence, the plates were rinsed twice with PBS to remove non-adherent cells. The cell monolayer was scratched with a pipette tip (10 ml) to generate 3 scratch wounds and was then rinsed twice with PBS to remove non-adherent cells. After 0 h and 24 h, the distance between the wound sites was measured. At least three independent experiments were performed for each condition.

### Statistical analysis

Continuous variables are expressed as means ± standard deviations and were compared using the student t test. Categorical data were shown as number(frequency) and were compared using the chi- square test. Multivariate analyses were conducted by the Cox proportional hazards model. Potential risk factors with P < 0.05 in the univariate analysis would enter into the Cox model and were further analyzed using step forward method. Survival analysis was performed using the Kaplan- Meier method and was compared using the log- rank test. The statistical analyses were performed using Prism GraphPad 8 or the SPSS statistical package (version 20.0; SPSS Inc., Chicago, IL, USA). A P value < 0.05 was considered significant.

The differentially expressed genes (DEG) between both groups were analyzed by the package edgeR in R software. The DEGs were identified on the basis of |log2(foldchange)| >1 and p < 0.05. DAVID was applied to perform the function enrichment and biological analyses of these DEGs. P<0.05 and gene counts ≥10 were considered statistically significant. The significantly enriched pathways were identified by comparing them to the Kyoto Encyclopedia of Genes and Genomes (KEGG) database, a database containing large-scale molecular datasets and used to explore the high-level functions of the biological system. The paired samples Wilcoxon test between paired ICC was analyzed using R package with PairedData. Correlation Analyses was performed using R package with Corrplot. The heatmap was plotted using R package with Heatmap. R software version 3.5.3 was devoted to statistical analysis and plotting.

## Supplementary Material

Supplementary Figure 1

Supplementary Table 1
