# Supplementary material for: Loss of FoxA2 accelerates neoplastic changes in the intrahepatic bile duct partly via the MAPK signaling pathway
Source: Aging (Albany NY). 2019 Nov 5;11(21):9280–94. doi: 10.18632/aging.102332 (PMC6874455; doi:10.18632/aging.102332)
Supplement: Supplementary Figure 1 [file aging-11-102332-s002.pdf]

SUPPLEMENTARY FIGURE

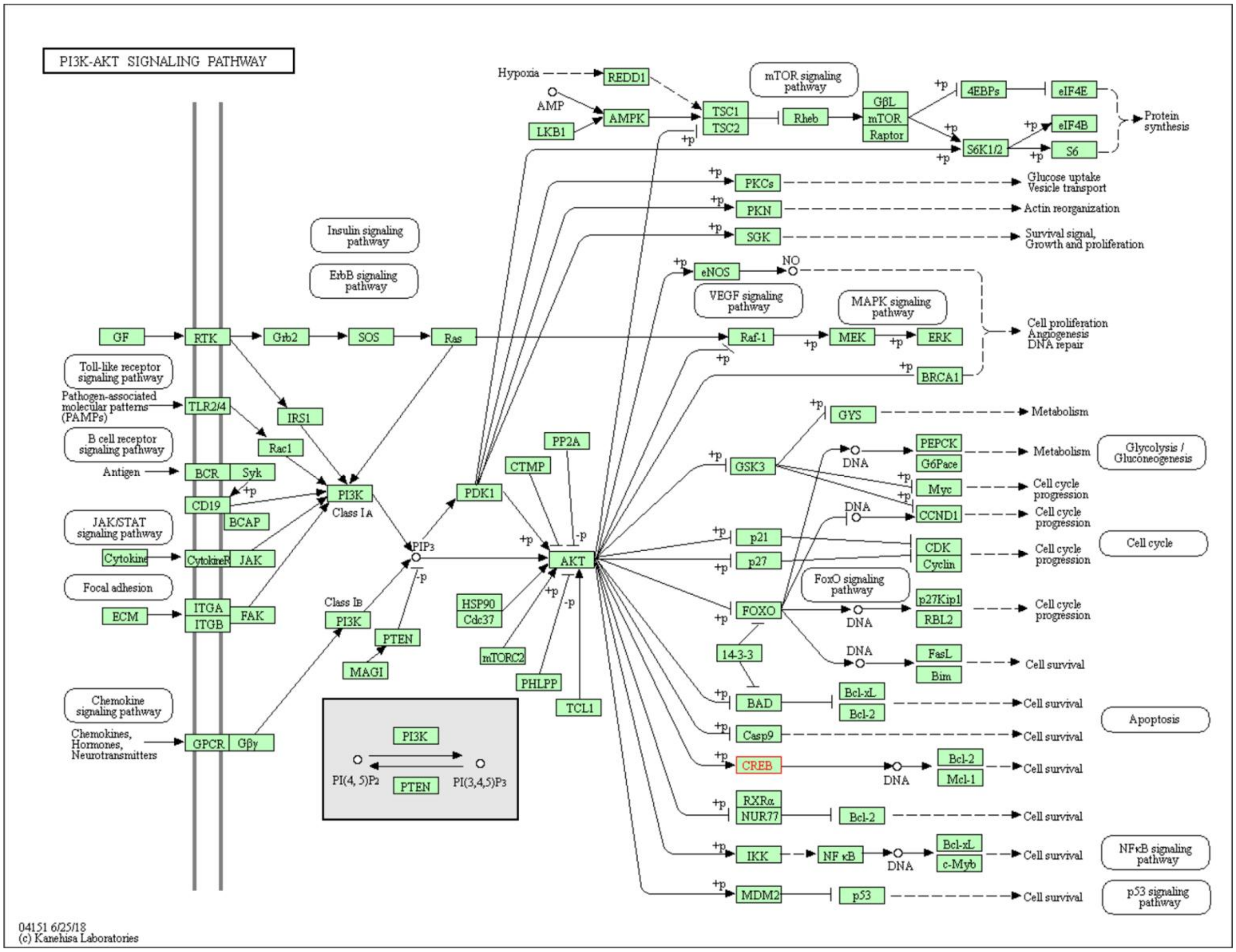

Supplementary Figure 1. AKT signaling pathway from KEGG database. CREB was regulated by activation of AKT.
