# Supplementary material for: Loss of FoxA2 accelerates neoplastic changes in the intrahepatic bile duct partly via the MAPK signaling pathway
Source: Aging (Albany NY). 2019 Nov 5;11(21):9280–94. doi: 10.18632/aging.102332 (PMC6874455; doi:10.18632/aging.102332)
Supplement: Supplementary Table 1 [file aging-11-102332-s001.pdf]

SUPPLEMENTARY TABLE

Supplementary Table 1. Genes.

genes
MAPK3
MAPK8IP2
MAPK12
MAP3K1
MAP2K6
MAPKAPK2
MAPK7
MAPK13
NRAS
HRAS
MRAS
FLNA
STMN1
PDGFA
PDGFD
IRAK1
EFNA4
HSPB1
CACNB3
CACNB1
NFKB2
RELB
PDGFB
AKT3
RAC1
CDC25B
EFNA5
TGFA
TGFB2
DUSP4
MECOM
EFNA3
IGF1R
CDC42
VEGFB
PAK1
TRAF2
STK3
PRKCA
TAOK1
ANGPT2
PPP5C
DAXX
KITLG
NF1
GNA12
TAOK2
